# Development of a theory and evidence-based program to promote community treatment of fevers in children under five in a rural district in Southern Ghana: An intervention mapping approach

**DOI:** 10.1186/s12889-016-3957-1

**Published:** 2017-01-25

**Authors:** Mercy Abbey, L. Kay Bartholomew, Margaret A. Chinbuah, Margaret Gyapong, John O. Gyapong, Bart van den Borne

**Affiliations:** 1 Research and Development Division, Ghana Health Service, PM Bag 190, Accra, Ghana; 2 School of Public Health, University of Texas Health Science Centre, 1200 Herman Pressler, Suite W238, Houston, TX 77030 USA; 3 Dodowa Health Research Centre, Ghana Health Service, P.O. Box 1, Dangme-West District, Ghana; 4 Present Address: University of Health and Allied Sciences, Ho, Ghana; 5 Department of Health Promotion, University of Maastricht, P.O. Box 616, Maastricht, 6200 MD The Netherlands; 6 Current Address: University of Health & Allied Sciences, Ho, Volta Region Ghana

**Keywords:** Intervention development, Intervention mapping, Community based program, Community health workers, Caregivers, Children under five, Ghana

## Abstract

**Background:**

This paper describes the development and implementation of a program to promote prompt and appropriate care seeking for fever in children under the age of five. Designed as a multicomponent program, the intervention comprises elements to influence the behavior of caregivers of children, Community Health Workers, professional health care providers and the wider community.

**Methods:**

Following the six fundamental steps of the Intervention Mapping protocol, we involved relevant stakeholders from the commencement of planning to program end. The IM protocol also recommends various behavior change methods to guide intervention development.

**Results:**

The intervention components implemented were successful in achieving program goals. For example, the intervention resulted in the primary outcome of reductions in all-cause mortality of 30% and 44%, among children treated with an antimalarial and those treated with the antimalarial plus an antibiotic respectively.

Most Community Health Workers were retained on the program, with an attrition rate of 21.2% over a period of 30 months and the Community Health Workers rate of adherence to performance guidelines was high at 94.6%.

**Conclusion:**

We were able to systematically develop a theory- and evidence-based health promotion program based on the Intervention Mapping protocol. This article contributes to the response to recent calls for a more detailed description of the development of interventions and trials. The intervention mapping approach can serve as a guide for others interested in developing community- based health interventions in similar settings.

**Electronic supplementary material:**

The online version of this article (doi:10.1186/s12889-016-3957-1) contains supplementary material, which is available to authorized users.

## Background

Mortality in children less than five years of age, remains an important public health problem especially in Sub- Saharan Africa (SSA) where almost half of the 7.7 million child deaths recorded globally occur each year [1–3]. Malaria and pneumonia alone cause about 1.4 million childhood deaths every year [4]. Effective treatment is available through interventions such as the use of antimalarials and antibiotics, but prompt diagnosis and treatment remain essential [5].

It is estimated that a 70% reduction in under-five mortality could be achieved if all cases of childhood pneumonia were managed at the community level [6]. Similarly, Community Case Management (CCM) of malaria can reduce overall and malaria-specific childhood mortality by 40% and 60%, respectively, and severe malaria morbidity by 53% [7]. Most developing countries with high mortality rates, have limited access to prompt and appropriate care for potentially deadly childhood diseases especially in rural areas [8]. Poor access contributes to caregiver’s failure to seek care and delays in seeking appropriate care which also contributes to a large number of child deaths [9]. Other reasons for delays in care seeking include geographic and financial barriers to access to health care facilities, poor knowledge and practice of caregivers and caregiver’s perceptions that health care providers have poor attitudes towards them [10–13].

Efforts to increase access to prompt and effective treatment of common childhood illnesses led to the introduction of community based interventions such as the Home Management of Malaria (HMM). Under this strategy children presenting with fever were treated presumptively with antimalarial drugs [14] usually by Community Health Workers (CHWs) who are lay persons selected by their communities and given basic training by professional health care staff to provide care for specific ailments. While this strategy expanded access to treatment of uncomplicated malaria; evidence showed misdiagnoses and inappropriate treatment of non-malaria febrile illnesses including pneumonia [15].

In 2004, the World Health Organization (WHO) and the United Nations Children’s Fund (UNICEF) supported recommendations for the integrated community case management (ICCM) of common childhood illnesses that included malaria, pneumonia, and diarrhoea [7]. Following the recommendation, the Ghana Health Service implemented a randomized controlled trial in the Dangme West district to operationalize the approach of involving CHWs in the management of pneumonia at the community level and to assess the impact of the intervention on under – five mortality in the study district [16–19]. To complement this community- based trial, we adopted a systematic approach [20] to guide the planning of a multi- component health promotion program. In this report, we describe the development and implementation process of our program.

Many authors have argued for a systematic approach for development and reporting of theory and evidence--based interventions [20–23]. Reporting in detail the design process and intervention implementation could promote the understanding of how effective interventions are made. Further, reporting in sufficient details could make it easy for other scientists and practitioners to replicate.

## Methods

### Planning method

We used Intervention Mapping (IM), a six- step tool for the systematic planning of health promotion programs [20] using theory, evidence from the literature and further research as needed to develop a behavior change intervention to promote prompt and appropriate care for febrile children aged 2 to 59 months in the Dangme West district in Ghana. IM has guided the planning and development of health promotion programs for many different health problems. Recent examples include cancer screening [24], smoking [25], obesity [26], chronic disease self-management [27]; sexuality, HIV and AIDS [28, 29], hearing loss [30], antiretroviral treatment adherence [31] and physical activity [32]. Others have explicitly used Intervention Mapping to guide community-based participatory research (CBPR) [33]; to better understand the active change ingredients in interventions [34, 35].

Each of the six steps of IM comprises several tasks. The completion of the tasks in each step leads to products that inform the subsequent step, and the completion of all steps creates a “blueprint” or “map” for designing, implementing, and evaluating an intervention. Step 1, entails describing the problem, the behaviours and environmental factors that cause the problem, among whom, and in what context. This first step of IM is based on the PRECEDE Model [36] and results in a simple logic model of the problem. In step 2, the specific behaviors and environmental conditions required to solve the problem are described, and their determinants explored to create a logic model of change. In step 3, planners choose theory-based change methods and practical applications matched to the behaviors and their determinants. In step 4, change methods are organized into a coherent, deliverable program leading to Step 5 where further work is done to ensure the program is feasible for communities to adopt and implement. Finally, in step 6, an evaluation plan is completed.

### Planning steps and procedures

#### Step 1: Formative research and logic model of the problem

In this step we defined the priority population and environmental change agents; reviewed reports of studies on community perceptions and care seeking for malaria and pneumonia in under- fives and the training, and use of CHWs. We also conducted field research including individual in-depth interviews with key informants, focus group discussions, and a survey of household caregivers of under- fives in the district [37]. We also solicited the perceptions of health professional staff on involving CHWs in the management of childhood fevers (to be presented elsewhere).

In the formative work, we defined a caregiver as that person whom household members perceived as having the primary responsibility of caring for an under-five child in the home. This person may or may not be the biological parent or blood relation of the child. In this paper, the words: Parent and caregiver are used interchangeably.

Our eight focus group discussions among household caregivers and eight individual In-depth Interviews with community key informants focused on perceptions, knowledge, behaviors and management of childhood fevers with emphasis on respiratory illnesses, (pneumonia) and care seeking. Details of these studies are presented elsewhere [37]. In brief, the focus group discussions involved 56 participants; Seven were held with females and one with male participants. Each group comprised seven members. All participants had at least one child under five. Most were married and had lived in their communities for at least 10 years. The women were predominantly traders and farmers and the men were engaged as farmers and artisans.

We defined a Key Informant as a resident community member acknowledged by peers as an opinion leader or a well-known community member having adequate knowledge to discuss issues pertaining to the inhabitants; including health issues and health seeking behaviour in the community. Key Informants comprised three Traditional Birth Attendants (TBAs), one herbalist, three chemical shop attendants and one community leader.

Following in-depth interviews and focus groups, we administered a questionnaire in a cross- sectional household survey among 501 caregivers to corroborate our qualitative findings.

The formative study was conducted as part of a larger study on home management of fever involving children aged 2 to 59 months and care givers in the Dangme West District. Based on data from the Demographic Surveillance System (DSS) in the Dangme West District- undertaken by the Dodowa Health Research Centre, study samples were selected through a random cluster sampling method for various surveys. Details of sample size determination and sampling strategy has already been reported elsewhere [38]. The respondents in the formative study were selected through a multi stage sampling technique reported in detail elsewhere [37]. In brief, a total of 60 communities were randomly selected and a list of all houses within those communities with children under-five was generated together with house addresses and other code names. With this list, interviewers visited the selected houses and interviewed only one care giver from one household within the house. If there was more than one eligible care giver, only one was selected for interview by simple random sampling.

Further, we distributed a self –administered questionnaire to professional health workers to assess their perceptions about involving CHWs in the management of childhood fevers. Purposive sampling was used to select respondents, who were responsible for treating children under five in the district health facilities. In all 15 out of a total of 20 eligible health workers who were available on the day of visit of the research team were included.

We organized IM Step 1 results in a logic model of the problem based on the PRECEDE model [36].

#### Step 2: Performance and change objectives

In step 2, we used the key findings of the formative study to define fever management behaviors for caregivers and CHWs, to describe determinants of the necessary behaviors and to develop a logic model of change (also called program or intervention theory). This step is based on the answers to the questions: “what do the participants of our program need to perform the health promoting behavior?”; “Why would the participants perform these behaviors?” The answers to the determinants questions were informed by behavioral theory with determinants including attitudes and other cognitions concerning the problem and the solutions. We then used the determinants and performance objectives to develop matrices of change objectives for caregivers, the wider community, CHWs and professional health staff in the district health facilities.

Working from the logic model of change, we developed matrices that combined behaviors with determinants to produce change objectives. These matrices were the foundation for decisions about what theory-based change methods (also referred to as techniques) [39] will likely influence change in determinants of the targeted behaviors in both the at-risk group and agents in the environment. A theory–based change method is a defined process by which theories postulate, and empiric research provides evidence for, how interventions can influence change in the determinants of behavior of individuals, groups, or social structures. Determinants of behavior almost always include many factors other than knowledge and awareness; therefore, methods must include processes to influence factors other than simple knowledge. Theory-based methods are likely to be a major foundation of an intervention’s *active ingredients* because they have been matched directly to the change objectives.

#### Steps 3 and 4: Change methods and practical applications for the intervention

In step 3 we identified theoretical methods for changing determinants specified in step 2 and selected delivery strategies and then organized these into a coherent program of practical applications (Step 4). For example, modeling with vicarious reinforcement, derived from Social Cognitive Theory [40] is an example of a theoretical method, which can be applied to increase skills and self-efficacy [20]. A practical application is the delivery of a change method or set of change methods in ways that fit the needs and preferences of the priority group and the context in which the program will be conducted. For example, to apply modeling, a program might include role model stories or skill demonstrations in person, in print, or in a mediated format such as video or computer. We added relevant methods to the logic model of change developed in this step.

#### Step 5: Adoption and implementation

In step 5, we planned for initial program use and testing of the program’s effectiveness. To increase the potential for program adoption, implementation and sustainability, we repeated the processes of considering needs, performance objectives, and determinants -- but this time for the program adopters, implementers, and maintainers . Thus, working with potential adopters and implementers, we reviewed the national implementation guidelines for integrated management of Childhood Illnesses, discussed the scope and training of CHWs and adapted the existing training materials for CHWs. Further, we planned for the orientation and role of health professional staff in the district as well as community involvement in the program.

#### Step 6: Planning the evaluation

In step six we planned for process and impact evaluation of the program by reviewing the logic model of change, developing evaluation questions and deciding on indicators and measures. We then planned an evaluation research design and data collection methods.

## Results

### Step 1: Formative research, needs assessment and logic model of the problem

**Fig. 1:**
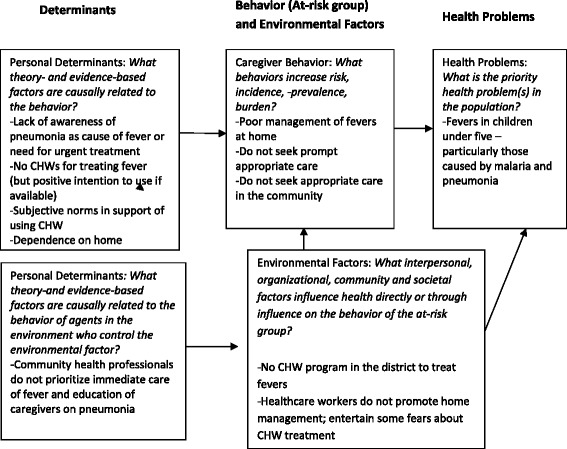
Logic model of the problem of delayed and inappropriate treatment of fevers in children under-five in Dangme West District

From the formative work, we developed Fig. 1, a logic model of the problem of delayed and inappropriate treatment of fevers in children under five. For example, studies report that caregivers often begin treatment at home and may include the use of a combination of herbs and or orthodox medicines, the use of inadequate doses of left-over medicines, the use of over-the-counter medicines and consultation with traditional healers. Care seeking in health facilities begins only after their initial treatments prove ineffective [41, 42]. Beginning on the right side of the model; we designated the main health problem to fever in children under five. Moving to the left in the model, the key caregiver behavior of concern is delayed and inadequate response to fevers in under-fives.

Factors contributing to this lack of appropriate response to fevers were found to be inadequate understanding by parents that fever can be related to *either* malaria or pneumonia and that both need urgent care. Caregivers reported that malaria is a serious disease, but the local language did not even have a word for pneumonia. The household survey also showed a high intention to seek care for childhood fevers from trained CHWs and perceived benefits and behavioral control (confidence) [43] about their ability to take a sick child to a CHW if available as well as positive subjective norms regarding the use of a CHW for childhood fever.

Health care workers also have a role in the inadequate response to fever by caregivers. They do not promote home management; nor do they seem to have much knowledge about the concept of home and community management strategies. Health care providers expressed some resistance to CHWs because they might detract from their professional practices and “popularity”. Some expressed the fear that CHWs might even ‘extort’ money from caregivers. Above all, the health workers were hindered by policies that privileged official interventions at the facility level for such illnesses. On the other hand, in community meetings, community members were relatively positive about the possibility of using CHWs for the management of childhood fevers. They wanted to know details such as how long medicines would remain available, who would pay CHWs, what type of training CHWs would have and whether they would treat other illnesses and other age groups above five years.

### IM Step 2: Change objectives

In this step, we converted the logic model of the problem to a logic model of change to depict health promoting behaviors and environmental conditions with their determinants and change methods. From this logic model of change, we developed matrices that combine performance objectives and determinants to provide the map for developing intervention strategies. Examples of partial matrices for caregivers, CHWs and health care providers are provided in Tables 1, 2 and 3. (Complete matrices are attached as Additional file 1)

**Table 1:** Partial matrix of change objectives for care givers of under- fives in Dangme West District Ghana

Performance Objectives The caregivers will:	Determinants (Specific targets of the communication)			
	Attitude	Perceived Social Norm	Perceived Control/skills Self-efficacy	Reinforcement
Seek prompt treatment from CHW when child has fever	Expect that new treatment and responding early will keep child from dying	Expect that husband or influentials will approve of going to the CHW	Describes confidence in taking action if child has fever	Health care providers make positive remarks about CHW treatment and referral
Go to CHW before acquiring any drug or herb or using leftover drugs	Expect that new drug will work to cure the child	Recognize that the community expects a good mother to respond immediately to a child who has fever by going to the CHW	Perceive that the recommended program has no cost at CHWs

**Table 2:** Partial matrix of change objectives for CHWs in Communities in Dangme West District

Performance Objectives for CHWs CHWs will:	Determinants (Specific targets of the communication)					
	Outcome Expectation	Subjective Norm	Perceived Control/skills Self-efficacy	Perceived severity knowledge	knowledge	Reinforcement
Greet and congratulate mother for coming immediately	Expect that caregiver will come to CHW if they are friendly in their approach		Describe how to greet and welcome the caregiver and specifically mention that she responded quickly		Explain that the first important task of the CHW is to make the caregiver feel welcome	Caregivers and community respect CHW
Assess child under 5 (and siblings who are presented at the same visit) and treat for fever	Describe how new treatment & responding early will keep child from dying	Expect their role in the community to be one of respect	Shows confidence in assessing child	Understand that child can die if action is not taken promptly for fever	Describe their role in the community as one that can help the children stay healthy and survive	Caregivers and community respect CHW

**Table 3:** Partial matrix for health professional staff in facilities in Dangme West District

Performance Objectives for health professional staff Health care providers will:	Determinants (Specific targets of the communication)				
	Attitude	Perceived Social Norm	Perceived Control/skills Self- efficacy	knowledge	Reinforcement
Express appreciation to caregivers that CHWs treat children with simple fever in the community. Compliment caregivers for promptly responding to referral by CHW	Express importance of CHW work Recognize importance of prompt reactions by caregivers by going to CHW if child has fever Recognize importance of attending promptly to the referred case	Recognize that other communities work with CHWs	Feel confident in treating CHWs as equal partners in treating fever in children at the community level	Understand that project is a research project to test the effectiveness of home and community-based management of fevers in children using these new drugs Describe the role of the CHW in community-based management of fever.	Praise caregiver for responding promptly to referral by CHW. Compliment caregiver for promptly going with child with fever to CHW

### Steps 3 and 4: Theory-based change methods and program development

In step 3 we used the logic model of change and the matrices to make final selections of theory-based change methods, practical applications, and delivery. We designed program materials to correspond to the parameters of use indicated in step 3 and reference to the change objectives in step 2. For example, we tailored the video delivered role model story to reflect the different participants of our program [40]. These included a coping role model female caregiver, a husband supporting his wife in taking their sick child to the CHW, a CHW being appropriate with rewarding behavior and a professional health staff praising caregivers for responding promptly to referral by CHW; in line with the social cognitive theory. In another example, the audiotaped message presented persuasive arguments about why to adhere to a prescribed treatment schedule and referral request. To ensure effective communication and enhance acceptability of the program messages, we were guided by the principle of cultural similarity [44] in selecting program implementers from the communities with similar socio-cultural background as the program beneficiaries. We pre-tested program materials among professionals as well as community members to ensure contents were technically correct and culturally sensitive.

The program comprised components to influence the behavior of four groups: parents/caregivers, CHWs, health professional staff, and the significant others in the community (See Table 4). For the parent/caregiver component, we delivered messages through videotaped drama, audiotaped messages via a public address (PA) system attached to our project vehicle and through oral presentations in face –to –face community meetings or durbars (Durbar is a gathering of community chief(s), elders and community members especially in rural areas, for important meetings). Theory- based methods used to influence determinants included modeling, persuasion, and vicarious reinforcement. Messages for those considered as significant others or opinion leaders in the community were delivered through the same channels as for caregivers and also through similar methods to influence the determinants. For CHWs, delivery was through educational video presentations (adapted from the IMCI training Video) and training activities. Methods used to influence determinants included modeling, skills training, information and demonstration and for the professional health care workers, we delivered messages through orientation meetings and to influence determinants; we used information dissemination, discussion, and modeling. Table 4 describes the change methods, delivery and sample messages for each component of the program.

**Table 4:** Selected examples of determinants, theoretical methods, and delivery for the four program components

Determinants (Theory Based Change Methods and Messages)	Program Materials, Practical Applications, and Delivery					
	Community Introductions (Durbars)	Videotaped Drama	Audiotape	Mobile Van	CHW training and protocol	Health Care Provider Orientation
PARENTS AND CAREGIVERS COMPONENTS						
Perceived Severity (Role Models, Persuasion, Instruction: fever can be a sign of severe illness – malaria and pneumonia and other illnesses)	√	√	√	√		
Attitudes (Modeling, arguments, persuasion: Take a child with fever immediately to the CHW. This can keep your child from dying).	√	√	√	√		
Subjective Norms (Role models, Persuasion: Husbands encourage your wives to take child with fever to CHW promptly)	√	√	√	√		
Knowledge (Modeling, information: CHWs are friendly)	√	√	√	√		
Perceived behavioral control/Skills What to do and how to do it (Modeling, information: Sponge child with fever, give paracetamol (if available) and take child immediately to CHW)	√	√	√	√		
Reinforcement (modeling, vicarious reinforcement, feedback: New drug from CHW is powerful, and it is free. friends and neighbors take child with fever to CHW for treatment)	√	√	√	√		
COMMUNITY MEMBERS						
Perceived Severity (Role Models, Persuasion, Instruction: fever can be a sign of severe illness – malaria, pneumonia and other illnesses)	√	√	√	√		
Attitudes (Modeling, arguments, persuasion: family members and neighbours encourage /support caregivers to take child with fever to CHW)	√	√	√	√		
Reinforcement (modeling, vicarious reinforcement, persuasion: CHWs work is voluntary, please show appreciation for services)	√	√	√	√		
COMMUNITY HEALTH WORKERS						
Reinforcement (Modeling, instruction: Sequence of CHW being appropriate with rewarding behaviour)		√			√	
Outcome Expectations( modeling, instruction: Caregivers will come to CHW if they are friendly in their approach)	√				√	
Subjective norms (Modeling, information: Health Care Providers, project staff and community members recognise the CHW role in the community to be one of respect)	√	√			√	
Skills/Self-efficacy(modeling, information: skills training, information: CHWs are well trained to assess sick child)	√	√			√	
Perceived severity (Modeling, instruction: child with fever can die if action is not taken promptly for fever)	√	√			√	
Knowledge (Modeling, Information: CHW role in the community can help the children stay healthy and survive)	√	√			√	
PROFESSIONAL HEALTH CARE PROVIDERS						
Attitudes (information, discussion, modeling: The role of CHWs in community management of fevers is important)						√
Self-efficacy (Information, Modeling: Health care workers are knowledgeable and self-efficacious professionals in working with CHWs in treating child with fever)						√
Reinforcement (Information, Modeling: Health care workers praise caregivers in responding promptly to referral by CHWs)						√
Knowledge (information, discussion: Health care workers discuss problems encountered in the work of CHWs that are not resolved during support visits, with project staff)						√

### Step 5: Adoption and implementation

#### Program implementation

Two different groups performed implementation activities: Community members implemented the community communication activities while personnel from the Ghana Ministry of Health conducted training for prospective CHWs. Four persons recruited from the communities and trained over two days implemented the community communications. The four persons, two per team, in consultation with community heads and opinion leaders, developed a schedule to cover date, time and an appropriate venue for the events.

The program implementers recruited participants through various means including using the community gong- gong beater (local town crier), the project vehicle with a public address system and by going door to door at households. Program delivery followed a standard routine. Typically, after observation of appropriate local protocol, (greetings, prayers, the introduction of team members, opinion leaders present and CHW(s) for the particular community), implementers gave a general overview of the project and an explanation of the meeting format. They then showed the video with interludes at the end of each scene for interaction with the participants to ensure learning objectives of the scene were achieved. The implementers showed the video at least once and sometimes twice in every community in the district where there was a working CHW. Meeting attendance ranged from 15–70 (depending on the size of the community). Community activities reached a total of 16,844 participants. These comprised 6,018 (35.7%) males and 10,826 (74.3%) females. Opinion leaders, including community chiefs, elders, executives of the local council, pastors and heads of organised women’s group attended the video sessions. The program Implementers played the audio messages at vantage points in the community from the project vehicle. To attract the audience, the team played popular music until enough people had gathered before the messages were disseminated.

### Community Health Workers

CHWs in groups of about 30 participants were trained for three days. Training was competence - and practice- based and was conducted in the local language. Trainers taught CHWs to assess sick children brought to them, provide treatment for those with uncomplicated fever, refer those presenting with signs of severe illness to health facilities and to counsel on medication and or referral compliance. A total of 660 CHWs graduated and were re-introduced to their communities. During the short ceremony of introduction, CHWs were presented with the necessary tools and supplies for their work in the presence of their community members.

### Step 6: Evaluation

We conducted process and outcome evaluation for components of the program using both qualitative and quantitative data collection methods. Detailed results of these evaluations are reported elsewhere and briefly summarized here:

The primary outcome of the trial indicated a reduction in all-cause mortality of 30% and 44% in children treated for uncomplicated fever with artesunate-amodiaquine (AAQ) alone and those treated with AAQ in addition to amoxicillin (AMX) respectively. The reduction was in comparison to children who received standard care that includes treatment at home, by traditional healers, from retail drug shops, or from the formal health sector. Though both results were significantly different in comparison to standard care, there was no significant difference between the one drug and two drug treatments [17].

For the CHWs, we examined attrition and retention among CHWs and its related factors. CHW attrition rates were abstracted from the project database and factors related to attrition analysed from focus group discussions conducted among CHWs, who remained until the end of the project. In summary, the study found a relatively moderate rate of attrition of 21.2% over the intervention period of 30 months [16]. This relatively low rate was attributed to the high level of community involvement in various processes of the intervention leading to high community approval and support. Reasons for attrition included lack of remuneration, a possible weak sense of social responsibility, and negative attitude of some caregivers.

In assessing the adherence of CHWs to guidelines for the management of fever in under-fives, we reviewed CHW documentation of sick child consultations over the trial period. The study showed that CHWs’ adherence to treatment guidelines was high 94.6%; whereas adherence to referral guidelines was inadequate and inconsistent [18].

Our post intervention community survey among 562 caregivers and focus group discussions among 84 caregivers assessed community utilization, perceptions and related factors of Community Health Worker services. The results indicated that a majority, 93.06% (523 /562) of caregivers had knowledge about CHWs in their communities. More than half, 59.4% of caregivers had used CHW services at least once for the management of fever in their under-fives. Further, caregivers who were exposed to the communication intervention were about four times more likely to use the services of the CHWs compared to those who were not exposed (OR = 3.79, 95% CI (2.62, 5.49)), ( *P* < 0.001) [19].

## Discussion

We described, in this paper, how we designed and implemented a multi component program, to influence the determinants of the behavior of caregivers of children under five, CHWs, professional health workers and community leaders regarding management of childhood fevers through theoretically based methods. These methods included modeling, persuasion and skills training; and strategies including video and audio recordings and training.

We applied the IM framework [20] which proved useful for the systematic development, implementation and process evaluation of our program. We used theory, evidence from the literature, formative research, and community participation in the design and implementation that led to the realization of our program goals. Whether the same goals would have been obtained without a systematic process is doubtful. As expected, the outcome of each step of IM provided the relevant materials that fed into the subsequent step. For example from the formative study results in step 1, we gained insight into the problem of a community knowledge gap on pneumonia as a potentially deadly disease and the seeming absence of a local name for it. Also the inadequate response to fever as well as the factors related to the problem at the individual and external levels.

In step 2, the results of step 1, supported with additional evidence from theories of behaviour, for example, the theory of planned behaviour [43] and social cognitive theory [40] were useful for providing information for developing the matrices of change objectives. We were guided by these first two steps in the selection of appropriate theoretical methods for behaviour change in step 3. In step 4, we translated methods into practical applications ensuring that they matched our various groups. For example to effectively employ modeling, we ensured that the necessary parameters of use were present [40]. For attitude change, we ensured messages were presented in a persuasive manner through arguments [20], Ch. 3). For steps 4 and 5, we ensured adequate community involvement in the development, pretesting and finalisation of programs and in step 6, we designed both outcome and process evaluation of the intervention. Though the steps are consecutive, we applied them in an iterative manner, moving backward and forward between the tasks and steps as we gained more information and insight [20] (Ch.1).

The formative study was useful for gaining more insight and an understanding of the factors associated with community perceptions, knowledge, and management of pneumonia. From the results of the formative assessments, it became apparent that our intervention or message focus should be on fevers rather than on pneumonia or disease specific messages; given that community knowledge about pneumonia was inadequate and further that they had no local name for pneumonia. Fever is a well-known common overlapping symptom for both malaria and pneumonia [45, 46] therefore it was appropriate to use “fever” in our program to represent both malaria and pneumonia or to refer to febrile illnesses in general; Moreover, the community was not required to diagnose the type of febrile illness at their level given that the two diseases of focus (malaria and pneumonia) are not easily distinguishable in the absence of appropriate diagnostic tools [45, 46].

Our use of audio/visual tape recorded messages and drama facilitated the coverage of a wider audience in a shorter time and with standardised messages than the commonly used health education strategy of to face – to –face oral presentations commonly used in the Dangme West communities. Oral deliveries are more prone to dilution by the implementers as compared with audio or video tape recorded messages. Further, the video shows were appropriate because its target audience captures those who did not own TV sets and could have missed out on the messages if they were aired by television stations. The video shows were not encumbered by lack of electric power in the rural communities and the shows facilitated immediate post video show audience surveys. Given the low literacy level among the caregivers, face to face interviews was better suited. The strategy of playing music via the mobile van also served as a form of entertainment and attracted many to gather around to listen and dance, making it easy to recruit participants and to sustain their interest for the activity.

We continuously involved relevant stakeholders in various aspects of the program, an approach that fostered a sense of ownership among the community. It also enabled us to obtain feedback on processes during program development and implementation. Working with a local drama troupe to produce the drama/video role model story ensured that the characters and issues were of cultural relevance and created identifiable (credible) characters dealing with familiar day to day issues. These processes involving the dramatisation of issues made it more acceptable to the community because they could easily relate to the people and the issues.

### Limitations

In adopting the systematic approach of IM, we found the process to be time consuming, though useful. Conducting the formative study, which was necessary to gain the needed information to adequately specify the problem with its contributing factors, took almost six months to complete the entire process. However, other studies have reported much longer durations, including 12 months and more [24, 47, 48]. Gans and colleagues emphasized that developing a culturally sensitive successful program requires dedication of adequate time and other resources by researchers for the formative stage [47]. Similarly, Suzuki and colleagues also found the application of IM to be complex and time intensive; none the less, they concluded that “the final program product was relevant and useful to its participants, and the extensive planning and assessment largely determined that success” [49]. In the formative study, we employed the use of a self –administered questionnaire to assess the perceptions of health care providers  about involving CHWs in the management of childhood fevers. Some well-known limitations of self-reported questionnaires include low response rates and the possibility that some questions will be misunderstood. We tried to minimise these by developing simple questions to aid respondent understanding and also hand delivering of questionnaires to the facilities instead of mailing them. This may have accounted for the impressive response rate of 100%. Out of a total of 20 eligible respondents, 15 who were present on the day of visit of the study team were included after consenting. Given that professional health Staff are sometimes cautious about the involvement of CHWs in health care provision; the self-administered questionnaire approach was preferred to ensure anonymity and confidentiality of the respondents.

## Conclusion

Through the application of IM, we were able to establish a logical planning process for the design, implementation, and evaluation of a community-based program for the management of childhood fevers in a rural district in Ghana. The systematic and detailed planning could account for the success in achieving our program goals. Thus, our description of the process provides insights that may be replicated by other scientists interested in planning theory and evidence -based interventions in community settings.

## Additional file

Additional file 1: Full Matrices. (DOCX 28 kb)
