# Supplementary material for: Development of a theory and evidence-based program to promote community treatment of fevers in children under five in a rural district in Southern Ghana: An intervention mapping approach
Source: BMC Public Health. 2017 Jan 25;17:120. doi: 10.1186/s12889-016-3957-1 (PMC5267456; doi:10.1186/s12889-016-3957-1)
Supplement: Additional file 1: — Full Matrices. (DOCX 28 kb) [file 12889_2016_3957_MOESM1_ESM.docx]

*Full Matrices attached as additional file:*

**Development of theory and evidence based program to promote community treatment of fevers in children under five in a rural district Ghana: An Intervention Mapping Approach**.

**Performance and change objectives for target groups**

**Caregiver matrix**

| **Performance objectives (What caregivers should do as a result of the communications)** | **Specific targets of the communications** | | | | | |
| --- | --- | --- | --- | --- | --- | --- |
|  | **Attitudes** | **Subjective norms** | **Perceived control/skills self-efficacy** | **Perceived severity** | **Knowledge** | **Reinforcement** |
| 1. Seek prompt treatment from CHW when you observe fever in child.  1.a. Go to CHW before acquiring any drug or herb or using leftover drugs  1.b. Give paracetamol if available and sponge child (while preparing to go to CHW)  1.c. Remind husband that service is free and close by  1.d. If mother can’t find CHW in a day, go to the health facility  1.e. If mother can’t find CHW when there is a danger sign, go to health facility immediately. | Expect that new treatment & responding early will keep child from dying.  Expect that new drug will work for more people/ for child  Perceive previous instruction good, but new better  Expect no adverse effects (no itching like chloroquine) from new drug | Expect that husband or influentials will approve of going to the CHW  Recognize that the community expects a good mother to respond immediately to a child who has fever by going to the CHW  Recognize that the community members expect that a mother responds to all fevers – not just one that is caused by malaria | Describes confidence in taking action if child has fever  Perceive that the recommended program has no costs  Perceives Proximity  To CHW  Express confidence how to administer drugs | Describe that children can die if action is not taken promptly for fever  Argue that fever is often a sign of a severe illness – malaria or other illnesses | Perceive that Health workers are friendly toward caregivers;  Know drug recommendation has changed  Know what to expect from CHW  Describes how to administer the drugs  Perceive provider as friendly  Health care providers reinforce mothers about CHW  Perceives New drug works  Perceive that old instruction was good but new instruction is better | HWs make positive remarks about CHW treatment & referral |
| 2. Express appreciation to CHW | Appreciate that the CHW is working without compensation |  |  |  | Understands that voluntary work of CHW is very important and needs to be recognised | Tells appreciation to CHW |
| 3. Monitor & respond to danger signs |  |  | Express confidence in monitoring and responding to danger signs | Perceives danger signs need immediate reaction and perceives that monitoring is important | Tell that the following danger signs need immediate reaction: child unable to feed/drink, vomits everything he/she drinks or eats, convulsion, very sleepy and hard to awaken etc. tells to monitor persistent diarrhea, persistent fever after 2 days of treatment, or other unusual signs of ill health |  |
| 4. Give all medication as instructed  4.a. Give all doses (do not stop when symptoms stop)  4. b. Replace dose if child vomits less than 1 hour after dose | Recognizes importance of giving all medication as prescribed even if symptoms have subsided |  |  | Understands that not taking all medications as advised will not heal the child |  |  |
| 5. Respond to CHW referral as soon as possible | Want to go to health facility if CHW refers | Mentions that partner and other influentials want caregiver to go to health facility if CHW refers. |  | Tells that not going promptly for referral will cause the child to get more sick and may die | Mentions consequences of delay in going to clinic |  |
| 6. Tell friends and neighbours to take child with fever to CHW |  | Expect that friends and neighbours will appreciate it when caregiver shares her positive experiences with help from CHW |  |  |  | Praise friends and neighbours who take child with fever to the CHW for treatment |

**CHW Matrix**

| **Performance objectives for CHWs**  **The CHWs will:** | **Outcome Expectations** | **Subjective norms/Respect** | **Skills/ Self-efficacy** | **Perceived severity** | **Knowledge** | **Reinforcement** |
| --- | --- | --- | --- | --- | --- | --- |
| Assess child under 5 (and siblings who are presented at the same visit) and treat for fever | Describe how new treatment & responding early will keep child from dying.  Expect new drugs to work better/work for more people than chloroquine.  Expect that their role in the community will help children survive  Perceive previous instruction good, but new better way to take care of fever | Expect their role in the community to be one of respect | Shows confidence in assessing child  Shows confidence in using assessment form | Understand that child can die if action is not taken promptly for fever  Perceive fever as a sign of severe problem | Describe their role in the community as one that can help the children stay healthy and survive  Describe how drug recommendation has changed  Describe how old drugs do not work for many of the people  Explain that fever can be caused by many diseases – not just malaria  Explain that an ARI with fever is a serious problem that often comes with malaria, sometimes by itself and should be treated |  |
| 1. **Greet and congratulate mother for coming immediately** | Expects that caregivers will come to CHW if they are friendly in their approach |  | Describe how to greet and welcome the caregiver by specifically mention that she responded quickly |  | Explain that the first important task of the CHW is to make the caregiver feel welcome |  |
| **2. Establish age of the child and ask mother what is wrong with the child** 2.a. Note age on form  2.b. Tick symptoms on form | Expect that the caregiver is a good and accurate observer of her child | Explain that effective CHWs listen carefully to the mother describe what is wrong with the child | Show on the form where and how to note age  Show on form the list of pictures with symptoms and how to check them off when the mother mentions them  Describe what each picture means  Expresses confidence in being able to begin discussion with caregiver |  | Explain that immediately after making the caregiver feel welcome, a CHW should find out what is wrong with the child by asking the mother |  |
| **3. Manage situation where child is over 5 years** |  |  |  |  | Knows to assess children over 5 as well as the under 5    Knows to provide medication if child over 5 needs it  Describes when to refer over 5s based on assessment |  |
| **3. If fever has not been mentioned, CHECK if there is no fever**  **3.a. If no fever REFER to health facility**  3.b. Tick referral on form/lack of fever | Explain that a program that helps caregivers to treat fever can help keep children from dying  Explain that for other problems parents will get good treatment at health facilities | Describe that effective CHWs treat fevers only  Describe that the community approves of the CHW program to treat fevers | demonstrate how to tick referral (when no fever) on form |  | Explain that the program is for children under 5 with fever because fever is dangerous in young children |  |
| **4. Check for danger signs by ASKING whether**  -Child is unable to drink or breastfeed?  -Child vomits everything?  -Child has convulsion during this illness (describes convulsion as unable to move arms and legs or loses consciousness)  -Child is hard to awaken, lethargic or unconscious  4.a. Tick danger signs on form | Expect that by listening to the caregiver’s report CHWs will be able to decide whether or not to refer child | Describe how effective CHWs take the danger signs very seriously | Demonstrate how to ask about the danger signs of not feeding, vomiting everything, unresponsiveness and convulsion  Expresses confidence in the ability to determine whether the child has had or is having danger signs | Explain that each sign an indicator of a condition that can be deadly if untreated | Explain what causes the signs |  |
| **5. Take history from mother by ASKING about**  -Duration of fever  -Whether child has a cough  -Duration of cough  -Whether child has difficult breathing  -Duration of difficult breathing  -Whether child has diarrhea  -Duration of diarrhea  -Whether there is blood in the stool  5.a. Tick presence or absence of  5.b. Note duration of symptoms on symptoms on form form |  |  | CHW feels self confident in asking and registering illness history |  | CHW should be able to list the different illness conditions for making a proper referral and providing proper treatment for symptoms |  |
| **6. Assess general condition of child by LOOKING for**  -Sunken eyes  -Crying a lot or irritable  -Generalized rash  -Pallor  -Swollen feet  -Hanging skin  -Abdomen pinch that recoils slowly  6.b. Tick general condition symptoms on form | Explain that a detailed record of the symptoms will help policy makers to know what problems children in the communities present with. |  | Express confidence in their skills to perform detailed assessment in general condition |  | Lists symptoms to look for in assessing general condition |  |
| **7. COUNT breathing rate**  7.a.Tick breathing rate on form |  |  |  |  | Names that breathing rate is counted for one minute and names the need for ticking on the form |  |
| **8. TREAT child with anti malarial drug only or anti malarial and antibiotic**  8.a. Tick treatment on form |  |  |  |  |  |  |
| **9. LOOK/observe first dose** |  |  |  |  | Mentions that observing taking of the first dose enhances compliance |  |
| **10. COUNSEL for drug adherence**  -Tell when to give medicine, how much and for how long  -Stress to give the drug until dosage is completed  -Tell mother if child vomits within one hour of taking medicine to replace the drug  10.a. Tick counseling for adherence on form | Tells that adequate counseling for compliance will ensure that treatment is effective |  | Expresses confidence in counseling about drug adherence including intake of all medication and what to do if child vomits | Lists the possible consequences if medication is not taken as prescribed | Mentions the importance of taking all fever drugs as prescribed even if child’s condition improves  Mentions the replacement of medication if child vomits |  |
| **11. If diarrhea, COUNSEL for oral rehydration therapy**  -ASK whether mother has started  -EXPLAIN and SHOW what liquid to give  11.a. Tick rehydration counseling o n form | Mentions that treatment for diarrhea is important for the survival of the child |  |  | Lists that persistent diarrheoa can cause death of child if action is not taken promptly | Lists what to describe how to feed and treat child with diarrhea |  |
| **12. COUNSEL mother to continue feeding child (if child will not eat, to give some sugary food or drink)**  12.a. Tick feeding counseling on form | Explain that a well fed child has better resistance to illnesses |  |  |  |  |  |
| **13. REFER caregiver to take child to health facility if any of the following were noted:**  -Inability to eat anything at all  -Vomiting everything  -Convulsions  -Lethargy/ unresponsiveness  -Diarrhea 5 days or more  -Fast breathing  -Chest in-drawing  -Stridor  -Pallor  -Swollen feet  -Slow skin pinch recoil  13.a. Tick referral on form |  | Perceive that project staff and health workers think its important to note referral on form |  |  | Explains that its important to refer child to health facility if the following symptoms are present:  -Inability to eat anything at all  -Vomiting everything  -Convulsions  -Lethargy/ unresponsiveness  -Diarrhea 5 days or more  -Fast breathing  -Chest in-drawing  -Stridor  -Pallor  -Swollen feet  -Slow skin pinch recoil |  |
| **14. COUNSEL what to do if child develops a danger sign at any time or doesn’t get better after 2 days**  14.a. Tick danger sign counseling on form |  |  | Demonstrates what to tell care givers what to do if danger signs are still present after 2 days |  | Names what to tell and how to motivate the care giver if one or more danger signs are still present after 2 days of visiting CHW |  |
| **15. COUNSEL caregiver to return after 2 days**  15.a. Tick counseling for when to return on form |  | Recognize expectation of program staff and health workers that reporting by care giver after 2 days is important |  |  | States that care givers must be told to return to CHW after 2 days for reporting on situation of child |  |
| **16. Asks when mother is able to go for referral**  -If soon, CHWs congratulates mother  -If mother indicates delay, CHWs stress the urgency  16.a. Tick referral planning on form |  |  | Demonstrates how to gently ask caregiver about referral plan |  | CHWs recognize the importance of reinforcing caregivers’ prompt action on referral |  |
| **17. Request mother to tell others to bring children with fever** | CHW realizes that telling others to bring child with fever will create community awareness and increase the number of children receiving prompt and appropriate treatment |  | Demonstrates confidence in telling other care givers what to do if their child has fever |  |  |  |
| **18. Maintain confidentiality** | CHW recognizes that maintaining confidentiality will increase the confidence of caregivers in CHW |  |  |  | Name why it is important to maintain confidentiality |  |
| **19. Position child if convulsing** |  |  |  |  |  |  |
| **20. Maintain spare drugs in stock** |  |  |  |  | Names or mentions that replacements should be available in case child vomits drug within an hour of intake |  |
| **21. Tick form when caregiver returns with the child for follow-up**  21.a. Tick whether child is better or not,  21.b. Refer if not better |  |  |  |  | Knows importance of recording follow up visit by caregiver; and knows to refer if child’s condition has not improved |  |

**Health Care Providers’ matrix**

| Performance objectives (What health care providers should do as a result of the communications) | Specific targets of the communications | | | | | |
| --- | --- | --- | --- | --- | --- | --- |
|  | Attitudes | Subjective norms | Perceived control/skills self-efficacy | Perceived severity | Knowledge | Reinforcement |
| 1.Health care workers express appreciation to caregivers that CHWs treat children with simple fever in the community  2.Health care workers compliment caregivers for promptly responding to referral by CHW  3. health care workers  - pay support visit to CHWs  - enquire about how work is going,  (Assessment, treatment, referral, record keeping, stock levels of drug, adverse effects, etc)  - discussing problems in the CHW work and solutions  4. health care workers inform project staff of problems related to CHW and discuss solutions and what needs to be done and by whom | Express importance of CHW work  Recognize importance of prompt reactions by caregivers by going to CHW if child has fever  Recognize importance of attending promptly to the referred case  Mention the importance of support visits to CHW and enquiring and discussing about the work  Express importance of reporting and discussing with project staff |  | Feel confident in treating CHWs as equal partners in treating fever in children at the community level |  | Understand that project is a research project to test the effectiveness of home and community based management of fevers in children using these drugs  List the role of the CHW in community based management of fever.  List the process of assessing, treating and referral of a sick child by CHW.  List their responsibilities and activities during support visit to CHWs  List what to do if problems encountered in the work of CHWs is not resolved during support visit | Praise caregiver for responding promptly to referral by CHW.  Compliment caregiver for promptly going with child with fever to CHW |
